# Large Scale Loss of Data in Low-Diversity Illumina Sequencing Libraries Can Be Recovered by Deferred Cluster Calling

**DOI:** 10.1371/journal.pone.0016607

**Published:** 2011-01-28

**Authors:** Felix Krueger, Simon R. Andrews, Cameron S. Osborne

**Affiliations:** 1 Bioinformatics Group, The Babraham Institute, Cambridge, United Kingdom; 2 Laboratory of Chromatin and Gene Expression, The Babraham Institute, Cambridge, United Kingdom; Victor Chang Cardiac Research Institute (VCCRI), Australia

## Abstract

Massively parallel DNA sequencing is capable of sequencing tens of millions of DNA fragments at the same time. However, sequence bias in the initial cycles, which are used to determine the coordinates of individual clusters, causes a loss of fidelity in cluster identification on Illumina Genome Analysers. This can result in a significant reduction in the numbers of clusters that can be analysed. Such low sample diversity is an intrinsic problem of sequencing libraries that are generated by restriction enzyme digestion, such as e4C-seq or reduced-representation libraries. Similarly, this problem can also arise through the combined sequencing of barcoded, multiplexed libraries. We describe a procedure to defer the mapping of cluster coordinates until low-diversity sequences have been passed. This simple procedure can recover substantial amounts of next generation sequencing data that would otherwise be lost.

## Introduction

Next generation sequencing provides unprecedented volumes of data, and is now used routinely to assess global transcription patterns (RNA seq), chromatin modifications (ChIP seq), and nuclear architecture (3C seq), among other applications. The Illumina Genome Analyser IIx is one of a few widely-used next generation sequencing systems. It employs a solid-phase, sequencing-by-synthesis method, where the DNA library, flanked by adapter sequences, is seeded upon on a lawn of oligonucleotides that coats the surface of the lanes on a flow cell. Each attached DNA fragment undergoes multiple rounds of amplification to create a cluster of identical DNA fragments. At each sequencing cycle, a fluorescently-labelled base is incorporated into each fragment in the cluster, and images of the flow cell surface are captured [1]. Image analysis algorithms are applied during the first few cycles to identify the positions of individual clusters (first 4 cycles for SCSv2.5/GOATv1.5 and SCSv2.6/OLBv1.6 or 5 cycles for SCSv2.8/OLBv1.8), which are then monitored through subsequent cycles to generate sequence data; the ability to read sequence from a lane successfully is critically dependent on the ability to correctly map coordinates of the clusters. Since its commercialization, advances have been made to increase the output of the sequencing systems such that Illumina systems are now capable of sequencing tens of millions of DNA fragments in each of the eight lanes on a flow cell. This provides exceptional depth of coverage, and indeed, for organisms with small genomes and certain sequencing applications this provides coverage well in excess of that which is required.

Given this potentially surplus depth of coverage, and that sequencing costs still represent a significant expenditure, it is attractive to have the capability to combine the sequencing of multiple libraries in a single experimental lane. Such multiplexing can be achieved by placing unique identifying bases, called a barcode, within the adapter sequence of each individual library in the mixture [2]. For multiplexing to be effective, data from individual libraries need to be sorted during the data processing stage. While Illumina market a multiplexing kit, a more simplistic multiplexing strategy places the barcodes at the junction between the adapter and DNA library. This permits the barcode and DNA library to be sequenced in a single, continuous run. Barcoding in this manner has been reported [2], [3]. However, there are implications with this multiplexing in this manner. Firstly, template read-length is sacrificed in order to sequence the barcode, although the read length can be extended if required. Secondly, placement of barcodes at the junction between the sequencing adapter and library will result in low sequence diversity at the start of the resulting library.

Some next-generation sequencing applications introduce low-diversity in the initial bases of a library such that they appear similar to multiplexed libraries. For instance, libraries generated for the analysis of both genome-wide interactions (e.g. e4C seq) and reduced representation bisulphite sequencing rely upon restriction enzyme digestion to fragment the library and incorporate the sequencing adapters, leaving a partial restriction enzyme recognition sequence present at the beginning of all fragments within the library [4], [5], [6]. The impact of low-diversity in the initial bases of the library has not been reported.

Here, we describe how the presence of a low-diversity mixture of sequences during the cluster calling cycles interferes with the mapping of cluster coordinates, and can result in a significant loss of data. Both the degree of diversity in the initial sequences and the cluster density on the flowcell impacts the extent of data loss. However, we find that by deferring the cluster coordinate mapping until the sequencing cycles that immediately follow the initially biased sequence, a maximal number of clusters can be identified. Furthermore, these cluster coordinates can still be used to determine the initially biased sequence. This simple, yet effective approach can dramatically increase the volume of data returned from libraries with a high degree of bias within the initial bases.

## Results and Discussion

**Figure 1:**
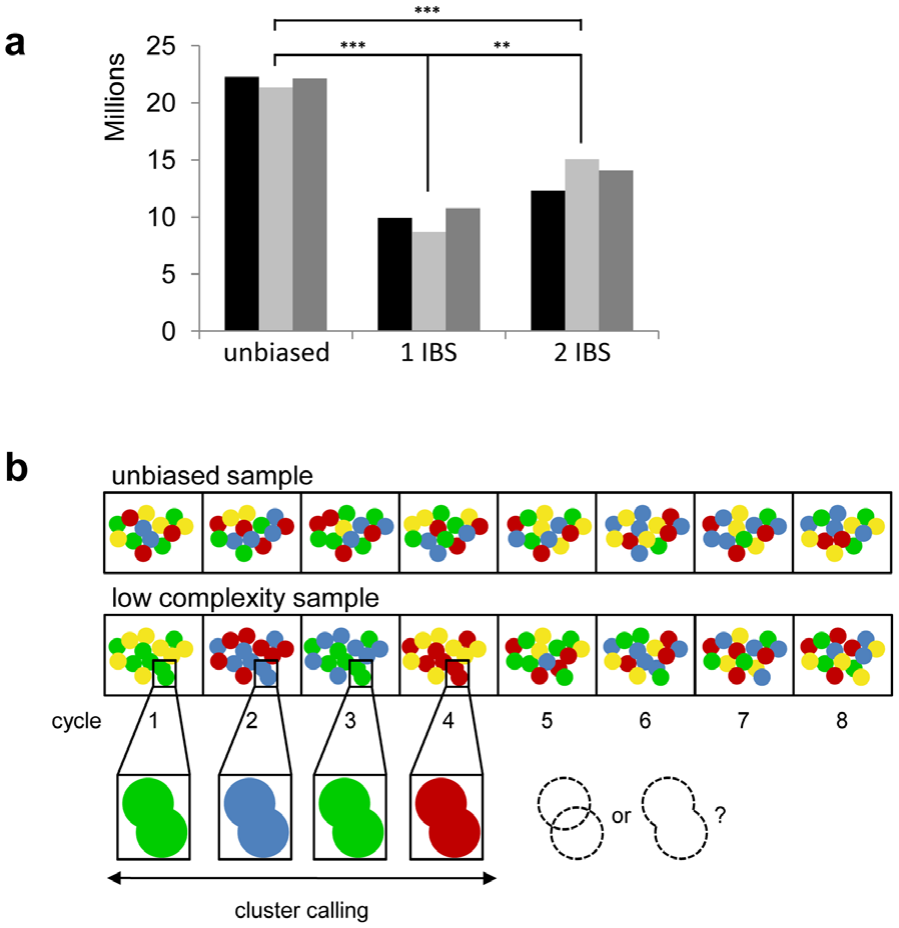
Fewer clusters are identified by the Illumina purity filter in low-diversity samples. (a) The total number of sequences per lane passing the purity filter step for samples with no bias, or one or two initially biased sequence (IBS) libraries. Data from three representative flow cell lanes are shown for each. (b) Current cluster-calling algorithms can discern clusters even at extremely high densities if the sequence composition during the first four cycles is unbiased. In low-diversity samples clusters with the same initially biased sequence in very close proximity may erroneously be called as a single cluster and may ultimately be removed by the purity filter.

We prepared Illumina sequencing libraries using custom-designed adapters that place a unique, four-base barcode sequence at the junction between the adapter and template. Thus the barcodes are sequenced during the first four sequencing cycles, immediately before the template. We combined equimolar amounts of libraries with unique barcodes to load into the same lane of a flow cell for sequencing. Compared to libraries that contain an unbiased initial sequence, we noted that libraries that contained a single barcode, or a mixture of two barcodes yielded significantly fewer sequences (Fig. 1a and Table 1). However, analysis of a sequencing lane that contained four barcoded libraries was not significantly different to unbiased libraries (not shown). We visually inspected image files from both barcoded and unbiased sample lanes to assess the relative densities of clusters, but could detect no discernible differences (not shown). Therefore, differences in cluster densities could not account for the discrepancies in sequence data volumes.

**Table 1:** Sequence yield of libraries with varying degree of initial sequence bias.

	exp 1	exp 2	exp 3	mean	% of 0 IBS
**0 IBS**	22261320	21343080	22150200	21918200	100
**1 IBS**	9934316	8712575	10774206	9807032	45
**2 IBS**	12291694	15086179	14057537	13811803	63

IBS: initially biased sequence; exp: independent experiment. Library origin was: 0 IBS -mouse, unbiased; 1 IBS - mouse, e4C; 2 IBS - yeast, 2 barcodes.

These observations indicated that the presence of the initially biased sequence may interfere with the identification of individual template clusters. We reasoned that algorithms designed to map the coordinates of template clusters may fail to distinguish two or more clusters with the same barcode when they are in very close proximity (Fig. 1b). Clusters can be rejected by the Illumina purity filter either due to their unusually large size or, once the sequences of these individual clusters diverge after the barcode, due to the presence of mixed sequence signals.

The base-calling algorithms associated with the Illumina sequence control software (SCS) are carried out in real time, which obliges cluster identification to be performed during the initial sequencing cycles. However, it is possible to re-analyse a completed sequencing run starting with the raw image files by invoking the GOAT pipeline (General Oligo Analysis Tool). This supports cluster identification and base-calling that begins at a later cycle. We wished to test whether the data output could be increased by deferring the cluster identification until after the barcode sequence.

**Figure 2:**
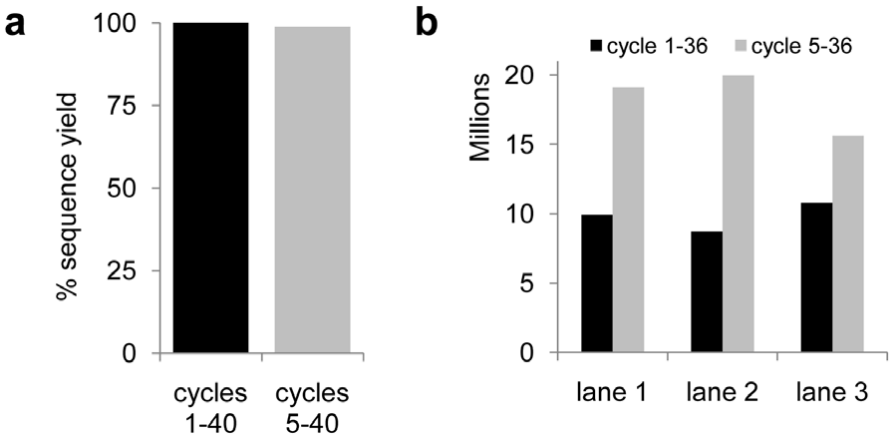
Deferred cluster-calling increases data output of low-diversity libraries dramatically. (a) The total number of sequences obtained from an unbiased PhiX control library processed with the SCS and GOAT pipelines. The number of reads returned by SCS analysis, commencing in cycle one was set to 100% and compared to GOAT pipeline-reprocessed raw image files, commencing in sequencing cycle five. (b) GOAT pipeline image analysis of three sample lanes with one initially biased sequence (IBS), comparing the number of sequences returned by analysis commencing in sequencing cycles one and five.

We first compared the cluster identification analysis of a sequencing lane containing the unbiased Illumina PhiX control library, using SCS and the GOAT pipelines. Whereas the SCS began its analysis in the first cycle, we configured the GOAT pipeline to start in sequencing cycle 5. There was virtually no difference in the quantity of sequences obtained, suggesting that the cluster identification algorithms are comparably efficient (Fig. 2a). Next, we carried out a similar analysis on three sequencing lanes, each containing a mouse e4C library with an identical initial sequence. Here, we observed a marked increase in the number of clusters passing the purity filter using the GOAT pipeline after starting the analysis from cycle 5. The data output in all three cases increased between 45 and 130%, eventually yielding nearly 20 million sequences per lane, close to the normal yield of 22 million sequences that is typically achievable using the SCS pipeline version 1.5 (Fig. 2b). There were no significant differences in the recovery of sequences from different initially biased sequences within each library (data not shown). Therefore, it appears possible to substantially increase the data output by reprocessing the image files starting after the barcode sequences.

**Figure 3:**
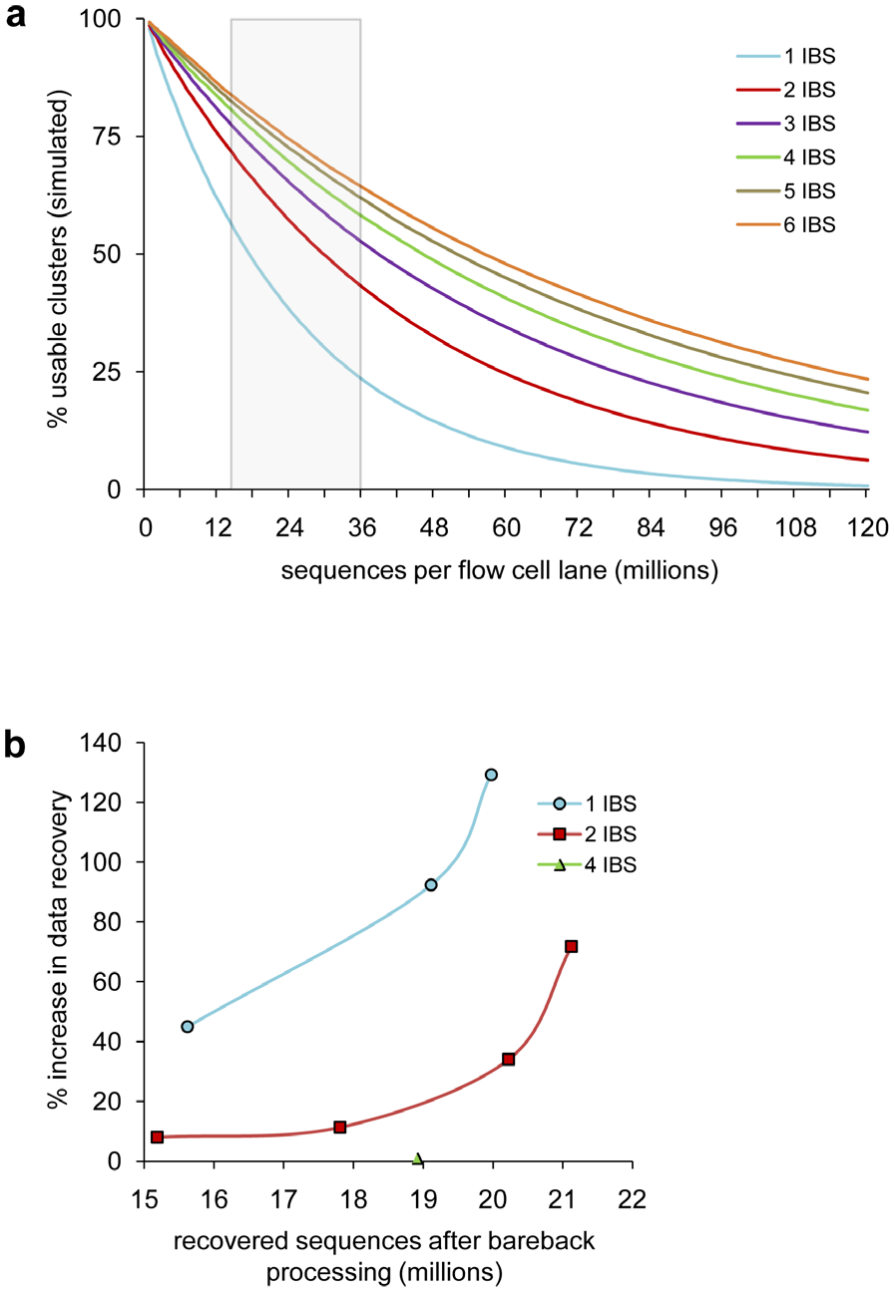
Diversity- and cluster density-dependent data loss can be avoided by bareback-processing. (a) Simulated effect of increasing cluster densities on the number of usable sequences for samples containing varying amounts of low diversity (one to six initially biased sequences, IBS). The grey box marks a currently sensible range of cluster densities (equating to 125,000–300,000 clusters per tile on a GAIIx). (b) Percent increase in sequence data obtained by bareback-processing in relation to cluster density; libraries contained either one (n = 3), two (n = 4) or four (n = 1) different IBS tags.

We hypothesized that the percentage of clusters that pass the quality filters will be influenced by both the density of clusters on the flow cell, and the degree of diversity of the sample. We generated a model to simulate the influence of sequence diversity has on percentage of clusters that pass the purity filters (Fig. 3a). Indeed this predicted that purity filtering reduces the percentage of usable clusters as the cluster density increases, and that low-diversity libraries are affected to a greater degree than more complex mixtures.

We next sought a method to allow the use of non-barcode cycles to perform cluster identification, and yet still retain the information encoded by the barcode. We reasoned that cluster mapping carried out at later cycles could be applied retrospectively to the image files for the biased barcode cycles. To achieve this, we wrote a simple program called bareback (barcode back-processing) that renamed the image files associated with each sequencing cycle. The image files from the first four cycles, containing the barcode sequence, were renamed to place them at the back of the image stack (i.e. cycles 37–40), and cycles 5 to 40 were re-designated as cycles 1 to 36. This procedure was carried out using image series from four lanes, each containing two barcoded libraries, mixed in equimolar amounts. As before, analysis by the GOAT pipeline yielded a substantial increase in the data output, ranging from 8 to 72% more data, compared to analysis using the SCS real time analysis (Fig. 3b). Importantly, the sequence information from the barcodes was preserved, and could be used to separate the sequence data from the combined libraries. We also applied bareback processing to mouse e4C sequencing samples in which all clusters contained an identical initial sequence. Similar to the sample that contained two barcoded libraries, bareback processing increased data recovery in a cluster density-dependent manner. In fact an increase of 130% was observed for the most densely clustered sample. In contrast, a data recovery from a sample that contained an equimolar mixture of four barcoded libraries was only marginally increased, suggesting that samples with four or more barcodes are sufficiently complex to allow efficient cluster identification by the SCS analysis. Taken together, the empirical measurements and simulation both suggest that potential for data recovery is a function of cluster density and the diversity of the barcode sequences, with the greatest benefits occurring with densely-clustered single barcode libraries. While this extreme situation is unlikely to arise from multiplexing, it will occur in libraries prepared from reduced-representation bisulphite sequencing analyses, or e4C-type experiments, where all sequences begin with the same restriction site [4], [5], [6]. Significantly in one case, over 21 million sequences were obtained following bareback-processing, which is close to the normal number of sequences that is typically obtained. This suggests that few sequences are lost resulting from low-diversity libraries, if deferred cluster identification is used.

**Figure 4:**
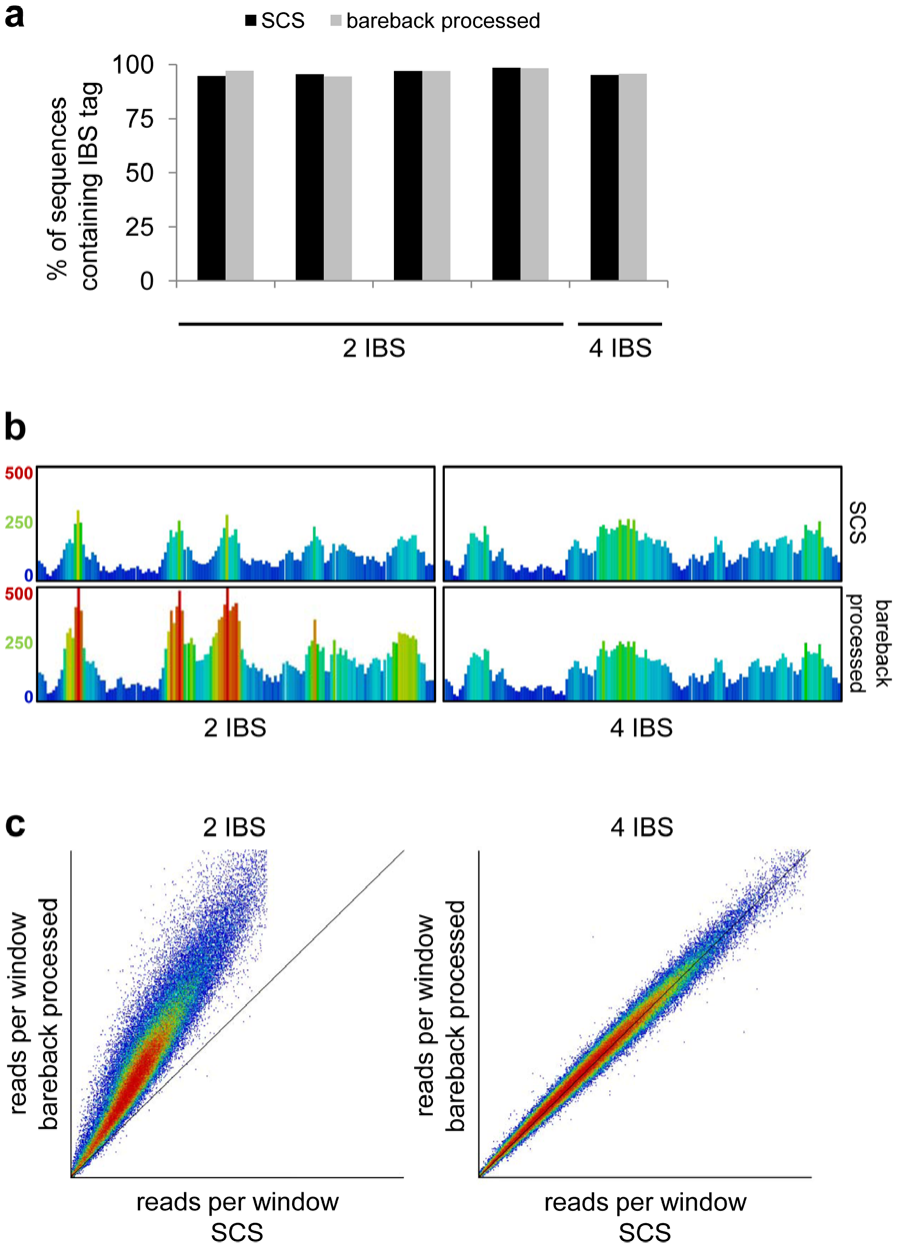
Bareback-processing increases sequencing depth without introducing bias. (a) The percentage of sequences containing the expected initially biased sequence (IBS) identifiers, processed with either the standard SCS pipeline or subjected to bareback-processing, shuffling the first four bp to the end of the sequence. (b) Comparison of the sequencing depth in a 14 kb region for two- and four-IBS library samples after SCS- or bareback-processing, followed by sequence alignment. Each bar represents a window of 100 bp, and the heat map colours range between 20 and 500 sequencing reads. (c) Scatter plot representation comparing the read count distribution of SCS- vs. bareback-processed samples with either two or four different IBS. Reads of an entire flow-cell lane were counted in sliding windows of 100 bp.

We investigated whether bareback-processing affected our ability to detect the barcode sequence in samples where the tag had been shuffled to the end of the sequence. In essence, the proportion of sequences with intact barcode stayed roughly the same in all samples analysed (Fig. 4a and Table 2), indicating that a net increase in sequence yield translates directly into an increased yield of barcoded, and therefore usable sequences. Also, we compared the SCS and bareback-processed samples after mapping the sequences to the genome. As expected, the number and pattern of sequences aligning to a randomly inspected region of the genome remained the same in the sample with four barcodes (Table 2 and Fig. 4b). In contrast, the increased number of raw sequences from bareback-processing of a sample containing only two barcoded libraries resulted in a much higher total number of mapped sequences, but with a similar genomic distribution (Fig. 4b). A genome-wide quantification of mapped reads generated by standard SCS or bareback-processing corroborates these findings (Fig. 4c). Thus, deferred cluster calling can be a valuable tool to efficiently achieve a higher sequencing depth for low-diversity sequencing libraries.

**Table 2:** Comparison of SCS and bareback-processed data.

	total sequence yield			reads containing expected barcodes					
				amount		percentage		%aligned	
	SCS	bareback	% increase	SCS	bareback	SCS	bareback	SCS	bareback
**2 IBS**	12291694	21125492	71.9	11647935	19369486	94.8	91.7	98.8	98.1
	15086179	20223717	34.1	14410499	19099795	95.5	94.4	96.5	96.1
	15994884	17808757	11.3	15490096	17270077	96.8	97.0	80.8	79.3
	14057537	15186074	8.0	13834946	14914810	98.4	98.2	95.7	95.6
**4 IBS**	18736285	18916427	1.0	17840122	18125796	95.2	95.8	95.7	96.5

Sequence data were obtained from barcoded *Saccharomyces cerevisiae* libraries.

Finally, an unofficial workaround to this problem has been reported to be included in the Illumina pipeline versions 2.6 and later [7]. While unsupported and undocumented by Illumina, the ‘image-flags’ option within the GOAT and SCS pipelines allows deferral of the cluster mapping to later cycles. In theory, this permits the sequence analysis to be conducted either in real time, or like bareback processing, once the run has been completed. We compared data processing using the ‘image-flags’ option with bareback processing, measuring both the data yield and sequence quality scores in unbiased and initially biased sequence libraries. We found that the sequencing data quality that was acquired by ‘image-flags’ was consistently poorer than bareback-processed data, as analysed by either per-base or per-sequence Phred quality scores (Fig. S1). In the case of one library that contained two initially-biased sequences, bareback-processing recovered four- to eight-fold more sequences than ‘image-flags’ (Fig. S1d-f). While we would not normally expect such a dramatic increase in data yield, based on our prediction of data loss for two initially biased sequences (Fig. 3A), we were able to fully rescue an otherwise completely failed Illumina run using bareback processing. It is surprising that ‘image-flags’ was unable to recover the same quantity or quality of sequences as bareback processing, and it is unclear how the methods differ.

Recently, Illumina has released a newer version of their pipeline, SCS v2.8/OLB v1.8, which promised an increase in cluster detection efficiency, and thus sequence yield. This new pipeline version uses the initial five cycles for cluster detection, rather than four in earlier versions, which may impact the efficiency of cluster identification. We compared the performance of SCS v2.6 with the latest version of the offline base-caller (OLB v1.8), using the standard pipeline, bareback and ‘image-flags’ processing on three biased sequencing libraries (e4C material). The new standard pipeline analysis was able to recover more sequences than the previous version, however recovery was generally poor (Fig. S2). Both bareback and ‘image-flags’ processing recovered considerably more sequences, yet again the sequence quality returned by ‘image-flags’ was worse than bareback. In summary, it seems that whatever improvements that have been made to the cluster identification algorithms are still insufficient to handle low-diversity library processing. However, this is overcome by bareback-processing, but not satisfactorily by the built-in image-flags option within the Illumina pipeline.

In summary, we have characterised inherent challenges in identifying the positions of clusters using low-diversity libraries for Illumina sequencing, and describe a simple, yet effective procedure to recover initially biased sequence data that otherwise would be lost through regular SCS pipeline processing. The use of multiplexing will become more widespread as the output of sequencers increases, and many applications will naturally generate biased libraries. Researchers who generate these types of libraries potentially can lose significant amounts of usable data unless they are aware of problems associated with biased libraries.

We demonstrate that back-processing of image data after a sequencing run is completed can recover lost sequence information, yet a preferred solution to this problem would be implemented in the real time sequencing software. Increasingly, Illumina sequencers are moving towards using real time analysis as their preferred or only analysis option, which necessitates that deferred cluster calling be implemented during the sequencing run. Using real time analysis presents three further challenges with respect to biased libraries: 1) The library must be known to be biased before sequencing is started, since there is no opportunity to reanalyse a library for which raw images were not stored. 2) Deferring cluster calling to later cycles requires raw data from earlier cycles to be stored on the processing machine until the cluster calling has been done and the full sequence analysis can commence. This could drastically increase the amount of local storage which processing machines would need. 3) Under the current implementation there is no facility to do cluster calling for different cycles in different lanes of a flow cell which would mean either filling a flow cell with similarly biased libraries, or deferring cluster calling on diverse libraries where this is not required.

The inherent problems associated with low-diversity libraries described here will also apply to the latest Illumina sequencing platform, the HiSeq 2000, since it makes use of the same chemistry and cluster detection algorithms as GAII systems. However, the HiSeq 2000, with its increased capacity and running costs, is designed to cater for specific niches of sequencing, such as shotgun and whole genome sequencing, which is less likely to encounter problems associated with low-diversity libraries. Applications that encounter initially biased sequences do not require the increased capacity of the HiSeq 2000, and are more likely to be sequenced on GAIIx and GAIIe systems, which continue to be marketed. For these applications, bareback processing is both feasible and highly useful.

In instances where deferred cluster-calling is not technically feasible, alternative strategies to maximize cluster calling in low-diversity samples can be employed. For instance, the diversity can be increased by using a mixture of different barcodes for each library within the sample. If the sequences of a sample are not intentionally barcoded, yet still contain a very biased sequence tag in the start, such as libraries generated from restriction digests, one could attach a short, random stretch of sequence to the start of the DNA fragments, as a means to artificially increase the sample diversity.

It is clear that low sample-diversity can potentially have a detrimental impact on the outcome of the sequencing run. Until Illumina has implemented a fix for this problem into their standard pipeline, careful adaptation of the experimental strategy, or the use of bareback-processing can both be valid approaches to tackle the problems associated with low-diversity sequencing libraries.

## Materials and Methods

### Library preparation

PhiX control and mouse methyl-DNA immunoprecipitation sequencing libraries were used as libraries without low-diversity initial sequence (no barcodes), and generated using conventional Illumina sequencing adapters with a 5′-T overhang. Libraries with a single initial sequence (one barcode) were derived from: a) mouse e4C sample that was digested with NlaIII, and annealed to a sequencing adapter that contained a 3′-GATC; and b) human e4C libraries, where the initial sequence tags were either TTTATTAAT, GGAATTAAT or TCGTTTATTAAT. Two- and four-barcode libraries were generated from *Saccharomyces cerevisiae* DNA, with the barcode sequences CATT, GTAT, ACGT and TGCT, using adapters as previously described [2].

### Sequence processing

Cluster-calling by SCS processing was carried out in real time, as part of the SCS v2.5 and 2.6 pipelines. Bareback-processing was carried out on the saved image stacks, using the GOAT pipeline v1.5 and OLB 1.6. A Perl script was generated to rename the image files for bareback-processing and is available for download (http://www.bioinformatics.bbsrc.ac.uk/projects/bareback/). For the analyses shown in Fig. S2, we used SCS v2.6 and the latest Illumina pipeline version, OLB v1.8.

### Alignment and mapping

Sequencing reads for two- and four-barcode libraries were aligned to the *Saccharomyces cerevisiae* genome (build SGD1.01) using Bowtie [8], run with the default options and -m 1, and mapped sequences were viewed in the SeqMonk genome browser (http://www.bioinformatics.bbsrc.ac.uk/projects/seqmonk/). Regions of 100 bp were sampled over the entire *Saccharomyces cerevisiae* genome, and only windows with a read count distribution between 0–99% were used; windows containing an abnormally high read count were excluded as they are most likely the result of mapping artefacts, rather than biologically meaningful.

### Statistical analysis

The sequence yields shown in Fig. 1a were analysed by ANOVA followed by Bonferroni post-hoc tests. It was assumed that the samples came from a normally distributed population and that the variability between the groups is approximately uniform.

### Cluster generation simulation

A cluster generation simulation was created for a single tile of an Illumina flowcell (dimensions 1888x2048 pixels), and results were extrapolated to an entire flow cell lane. Simulated cluster x- and y-coordinates were placed randomly on the tile at increasing densities and measurements were taken of the proportion of clusters which might realistically be expected to be resolvable upon subsequent image analysis. Simulated clusters whose centres were positioned within one imaged pixel of each other were always treated as non-resolvable. Those whose centres fell within 2.5 pixels of each other were rejected only if they shared the same sequence over the bases used for cluster calling. The simulation was run over a range of cluster densities and with a varying numbers of unique sequences in the cluster calling bases to assess the impact of reduced sequence diversity on the efficiency of cluster calling.

## Supporting Information

Figure S1 **The sequence recovery of bareback-processing can potentially recover vastly more sequences than the undocumented Illumina pipeline option “—image-flags.”** Three Illumina flow cell lanes containing libraries with different numbers of initial biased sequences (IBS) were processed with the standard real time analysis (SCS), the undocumented Illumina option “—image-flags” using either cycles 5-9 (—image-flags 5) or cycles 10-14 (—image-flags 10) for cluster detection, or using bareback processing (bareback). The sample sequences were either unbiased (a-c, PhiX control) or contained two IBS tags (d-f, two restriction enzyme ChIP-seq) or three IBS tags (g-i, reduced representation bisulfite-seq). (a, d, g) Average per-base quality score for all reads. Blue line: means, read lines: median, yellow box: 25 percentile, whiskers: 75 percentile. (b, e, h) Average quality score of all sequence reads. The graphs in (a-b, d-e, g-h) were generated with the quality control software FastQC, a quality control application for FastQ files (http://www.bioinformatics.bbsrc.ac.uk/projects/fastqc/). (c, f, i) Total sequence yield for each of the applied methods. These analyses were carried out using the Illumina CASAVA (SCS only) and OLB versions 1.6.
(PDF)

Figure S2 **Comparison of Illumina pipeline versions SCS v2.6 and v2.8 performance for very biased sequencing libraries.** A PhiX control lane (PhiX) and three Illumina flow cell lanes, each containing a single-barcoded human e4C library (lanes 1-3) were processed with standard real time analysis (SCS v2.6/RTA v1.6), or the latest version of the offline basecaller (OLB v1.8) for standard, image-flags or bareback analysis. (a) Total sequence yield for different analysis settings. Whereas processing of one-IBS libraries fails for both standard versions of the Illumina pipeline, the use of image-flags or bareback-processing (starting analysis from cycle 10 for Phi X and lanes 1 and 3, or cycle 13 for lane 2) recover a substantial amount of sequence data. Without saving images or using image-flags, the sequencing data would be irretrievably lost. (b) Average per-base Phred quality scores for each cycle of the sequence read (total read length 40 bp). Quality scores for libraries with or without initial sequence bias are consistently poorer for image-flags analysed data compared to bareback-processed data. (c) Total per-sequence Phred quality scores demonstrate a consistently higher quality of bareback-processed data. In addition to a higher proportion of low quality reads, image-flags analysed data contains up to 1.5 million reads with a Phred score of two throughout (quality value ‘B’); this special read segment quality control indicator implies that all of these sequences should be excluded from downstream analysis.
(PDF)
