# Supplementary figures and images for: Large Scale Loss of Data in Low-Diversity Illumina Sequencing Libraries Can Be Recovered by Deferred Cluster Calling
Source: PLoS One. 2011 Jan 28;6(1):e16607. doi: 10.1371/journal.pone.0016607 (PMC3030592; doi:10.1371/journal.pone.0016607)

Figure S1

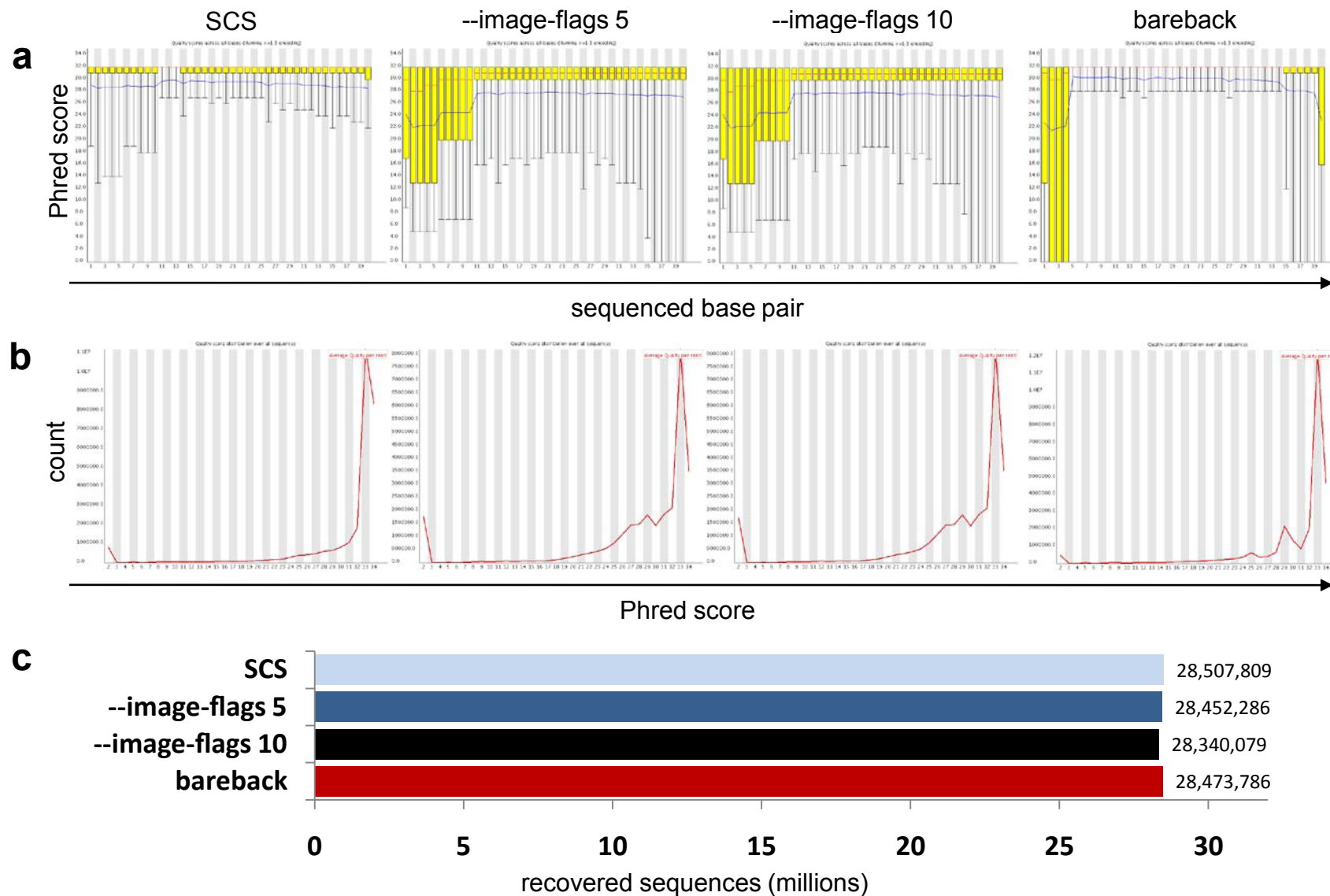

Figure S1

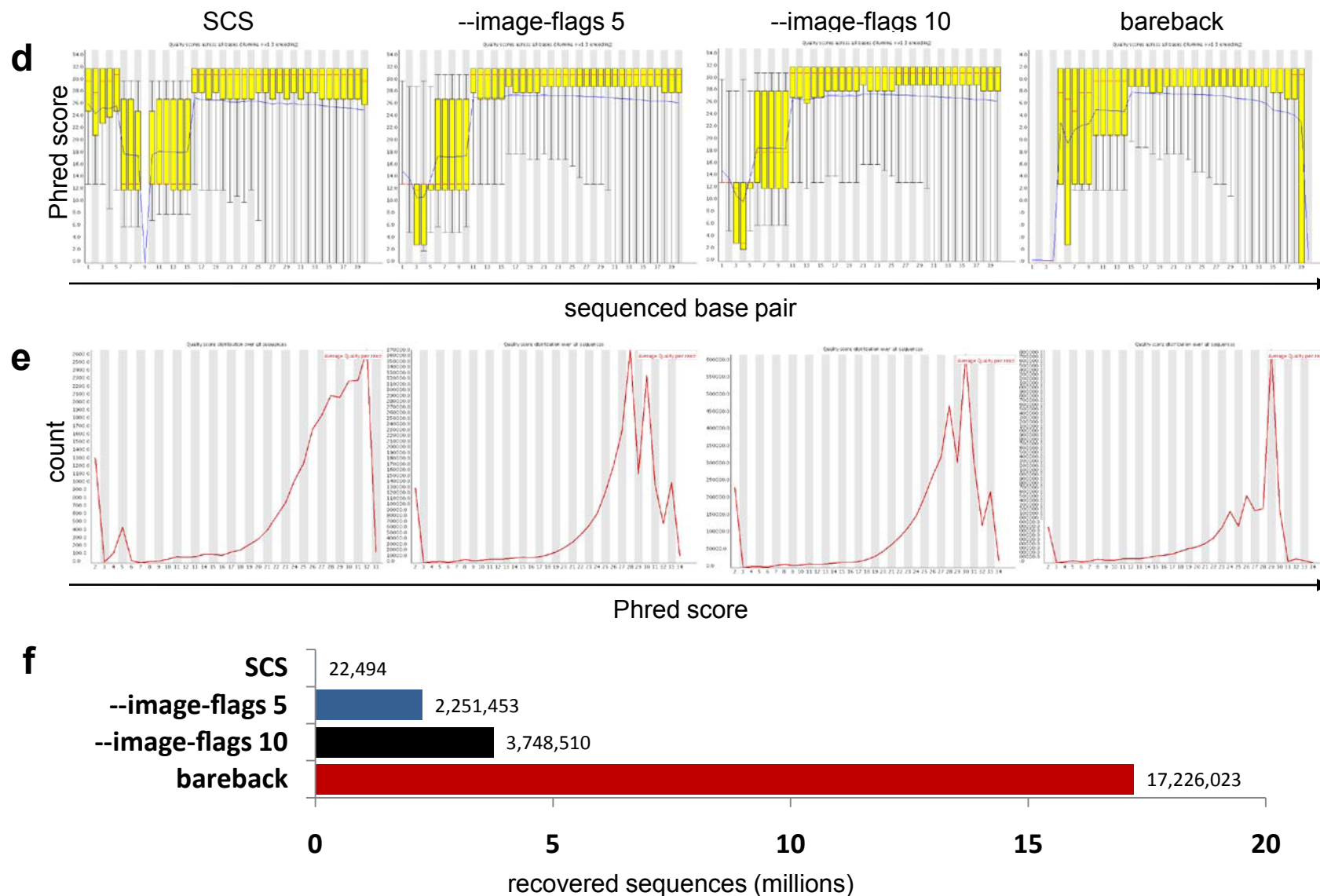

Figure S1

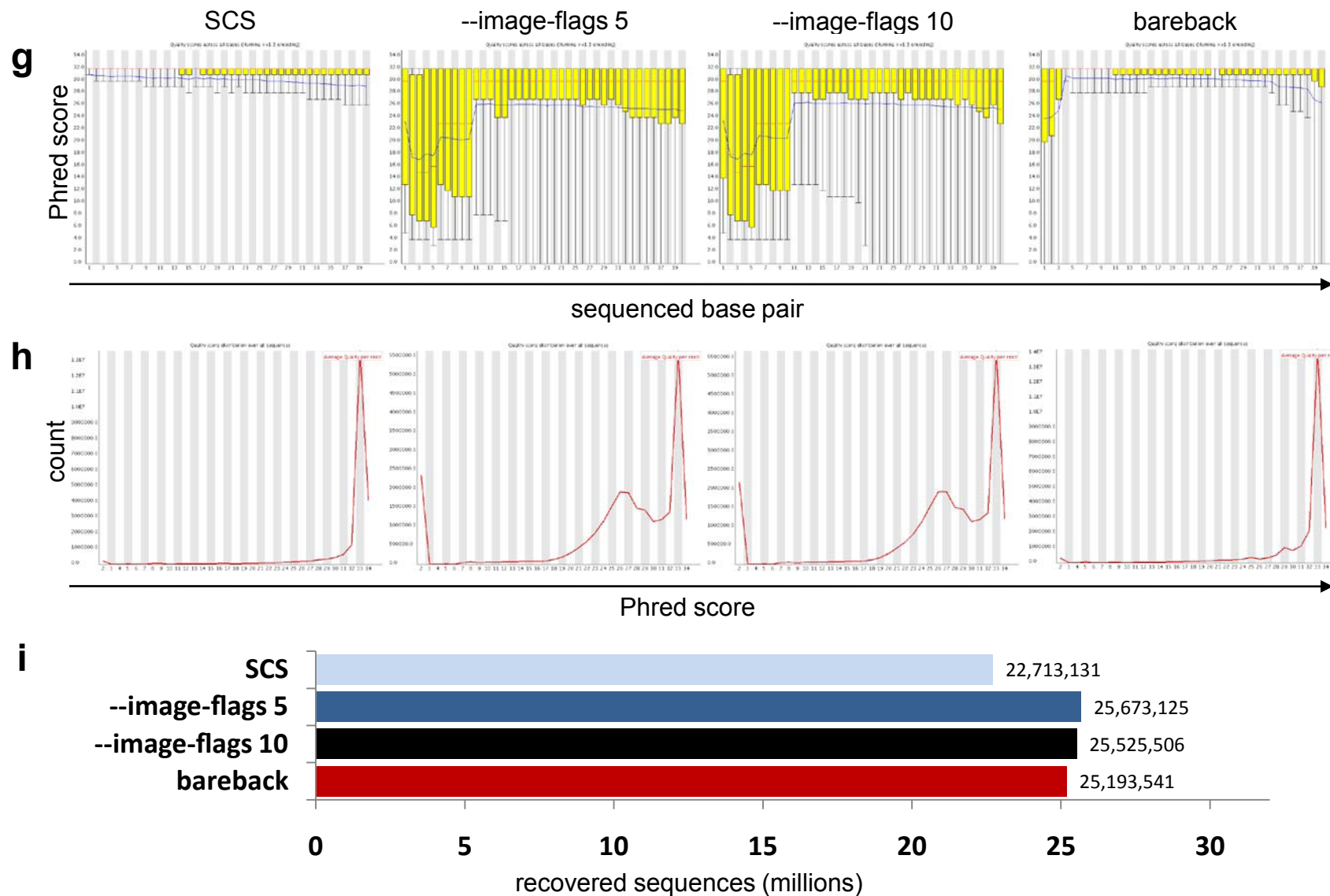

Supplement: Figure S1 — The sequence recovery of bareback-processing can potentially recover vastly more sequences than the undocumented Illumina pipeline option “—image-flags.” Three Illumina flow cell lanes containing libraries with different numbers of initial biased sequences (IBS) were processed with the standard real time analysis (SCS), the undocumented Illumina option “—image-flags” using either cycles 5-9 (—image-flags 5) or cycles 10-14 (—image-flags 10) for cluster detection, or using bareback processing (bareback). The sample sequences were either unbiased (a-c, PhiX control) or contained two IBS tags (d-f, two restriction enzyme ChIP-seq) or three IBS tags (g-i, reduced representation bisulfite-seq). (a, d, g) Average per-base quality score for all reads. Blue line: means, read lines: median, yellow box: 25 percentile, whiskers: 75 percentile. (b, e, h) Average quality score of all sequence reads. The graphs in (a-b, d-e, g-h) were generated with the quality control software FastQC, a quality control application for FastQ files (http://www.bioinformatics.bbsrc.ac.uk/projects/fastqc/). (c, f, i) Total sequence yield for each of the applied methods. These analyses were carried out using the Illumina CASAVA (SCS only) and OLB versions 1.6. (PDF) [file pone.0016607.s001.pdf]

Figure S2

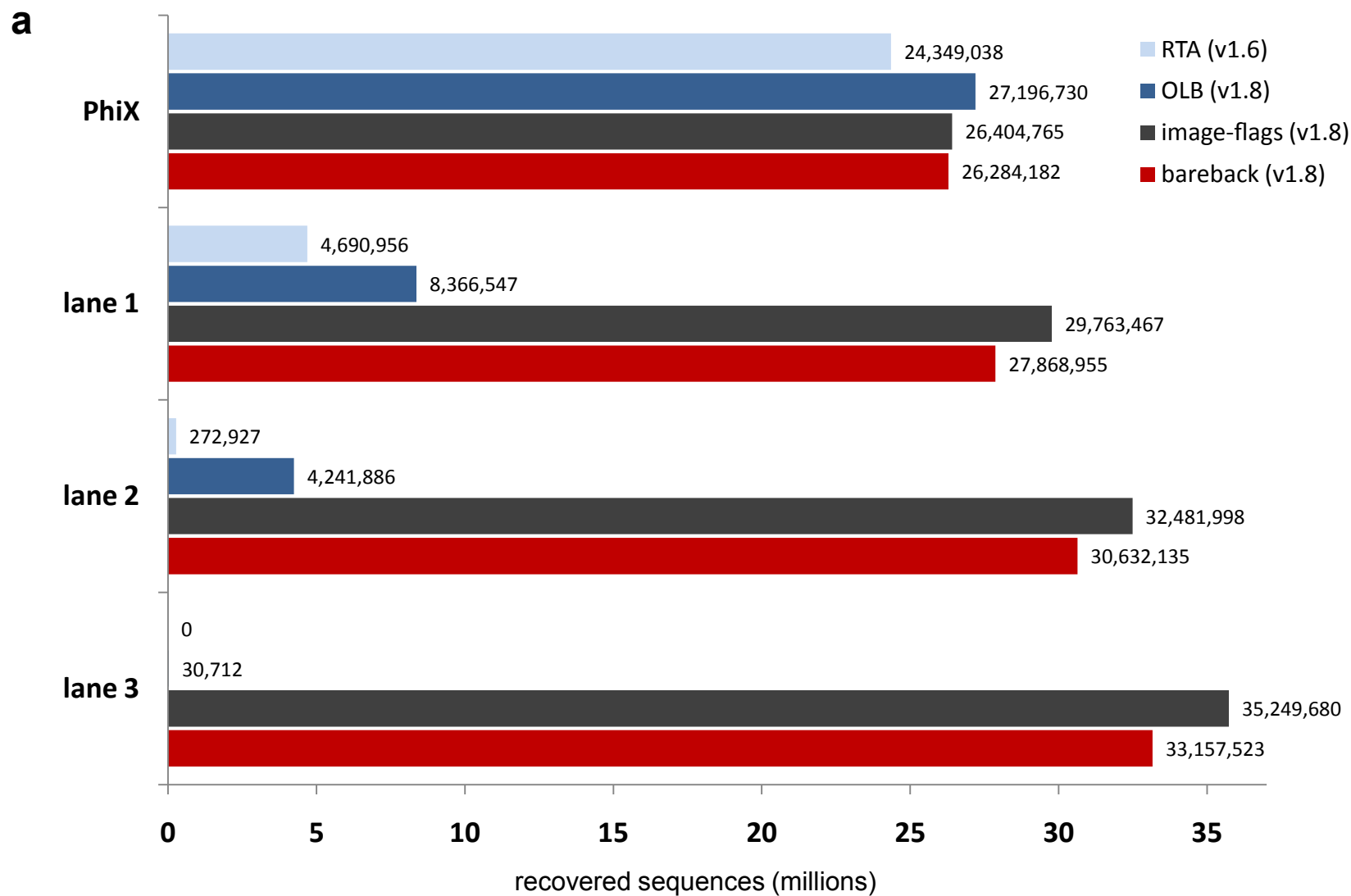

Figure S2

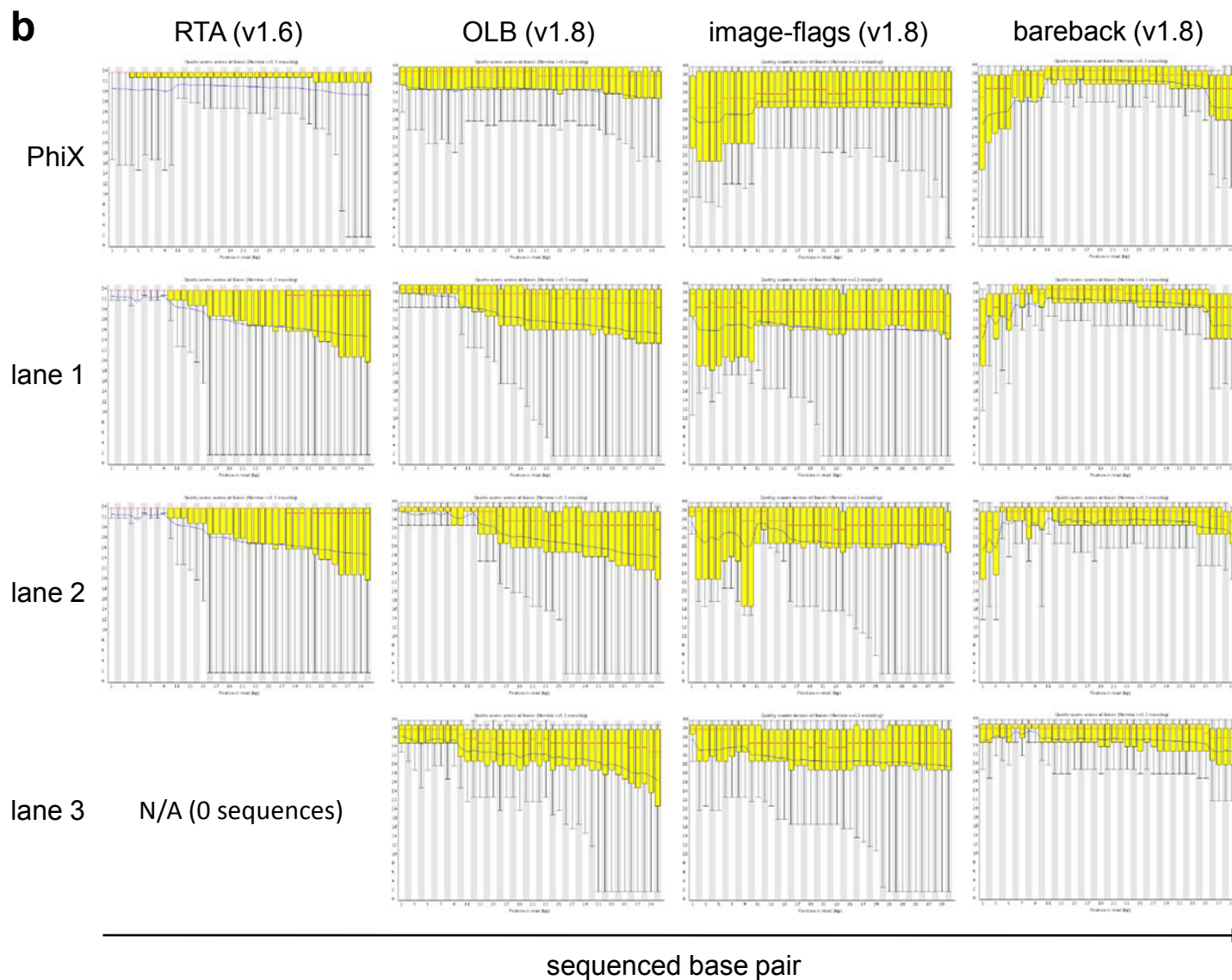

Figure S2

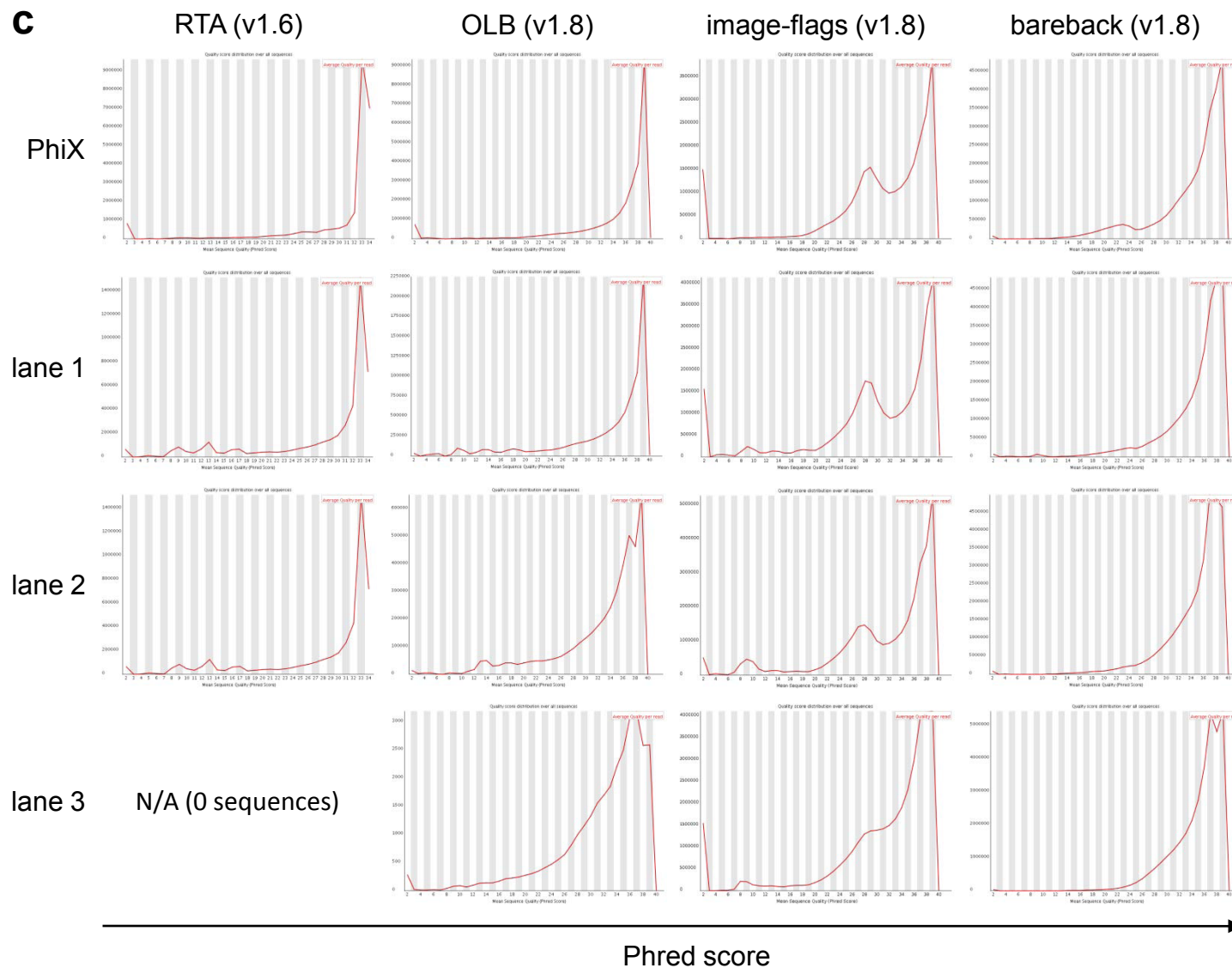

Supplement: Figure S2 — Comparison of Illumina pipeline versions SCS v2.6 and v2.8 performance for very biased sequencing libraries. A PhiX control lane (PhiX) and three Illumina flow cell lanes, each containing a single-barcoded human e4C library (lanes 1-3) were processed with standard real time analysis (SCS v2.6/RTA v1.6), or the latest version of the offline basecaller (OLB v1.8) for standard, image-flags or bareback analysis. (a) Total sequence yield for different analysis settings. Whereas processing of one-IBS libraries fails for both standard versions of the Illumina pipeline, the use of image-flags or bareback-processing (starting analysis from cycle 10 for Phi X and lanes 1 and 3, or cycle 13 for lane 2) recover a substantial amount of sequence data. Without saving images or using image-flags, the sequencing data would be irretrievably lost. (b) Average per-base Phred quality scores for each cycle of the sequence read (total read length 40 bp). Quality scores for libraries with or without initial sequence bias are consistently poorer for image-flags analysed data compared to bareback-processed data. (c) Total per-sequence Phred quality scores demonstrate a consistently higher quality of bareback-processed data. In addition to a higher proportion of low quality reads, image-flags analysed data contains up to 1.5 million reads with a Phred score of two throughout (quality value ‘B’); this special read segment quality control indicator implies that all of these sequences should be excluded from downstream analysis. (PDF) [file pone.0016607.s002.pdf]
